# Point-of-Care Ultrasound Education During a Pandemic: From Webinar to Progressive Dinner-Style Bedside Learning

**DOI:** 10.7759/cureus.25141

**Published:** 2022-05-19

**Authors:** Audrey Herbert, Frances M Russell, Gregory Zahn, Bita Zakeri, Christine Motzkus, Paul M Wallach, Robinson M Ferre

**Affiliations:** 1 Emergency Medicine, Indiana University School of Medicine, Indianapolis, USA; 2 Education and Continuing Medical Education, Indiana University School of Medicine, Indianapolis, USA; 3 Internal Medicine, Indiana University School of Medicine, Indianapolis, USA

**Keywords:** medical school ultrasound curriculum, bedside ultrasound, point-of-care ultrasound, clinical ultrasound, ultrasound

## Abstract

Objective: Point-of-care ultrasound (POCUS), traditionally, requires the proximity of learners and educators, making POCUS education challenging during the COVID-19 pandemic. We set out to evaluate three alternate approaches to teaching POCUS in UME. Sessions progressed from an online seminar to a remote, interactive simulation to a “progressive dinner” style session, as precautions evolved throughout the pandemic.

Methods: This prospective study details a series of three POCUS workshops that were designed to align with prevailing social distancing precautions during the COVID-19 pandemic. Overall, 656 medical students were included. The first and second workshops used web-based conferencing technology with real-time ultrasound imaging, with the second workshop focusing on clinical integration through simulation. As distancing precautions were updated, a novel “progressive dinner” technique was used for the third workshop. Surveys were conducted after each session to obtain feedback on students’ attitudes toward alternative teaching techniques and quantitative and qualitative analyses were used.

Results: The initial, remote POCUS workshop was performed for 180 medical students. Ninety-nine (177) percent of students felt the session was “intellectually challenging” and “stimulating.” Ninety-nine percent of students (340/344), after the second workshop, indicated the session was intellectually challenging, stimulating, and a positive learning experience. Students' ability to correctly identify pathologic images increased post-session evaluation from in-session polling. For workshop three, 99% (107/108) of students indicated that the session was “informative.” There was a significant improvement in pre- to post-workshop knowledge regarding image acquisition, interpretation, and clinical integration.

Conclusion: While image acquisition skills are best conveyed at the bedside, these modified POCUS teaching techniques developed and delivered in alignment with COVID-19 pandemic restrictions during a series of three workshops were shown to be effective surrogates for traditional teaching approaches when social distancing requirements, a large learner pool, or lack of local expertise exist.

## Introduction

The significance of point-of-care ultrasound (POCUS) in recent years as both a medical education tool and a valuable clinical assessment instrument has been increasingly realized [1]. Many medical schools use POCUS to support the understanding of anatomical relationships and to augment the comprehension of physical exam techniques [2]. As undergraduate medical education (UME) programs have realized the potential learning leveraged by POCUS and the consequent technical skills that translate into clinical care, many schools have incorporated this valuable instrument as a required component of their curriculum [3-6].

Performing POCUS is a technical skill that is acquired and perfected through practice. Teaching POCUS is conventionally performed with learners at the bedside, in close proximity to each other. Small group sessions are ideal for this type of learning and require an ultrasound educator, a small group of learners, and a model or patient. As POCUS has only been available in the last 30 years and only more recently popularized, ultrasound medical education during a pandemic has never been experienced [2]. Unlike most medical knowledge acquisition, ultrasound education cannot be fully delivered through didactics or textbook/online reading alone. Although basic POCUS concepts and interpretation do lend themselves to these educational techniques, hands-on image acquisition skills require demonstration and continual practice.

Due to the need for social distancing during the COVID-19 pandemic, traditional in-person POCUS training sessions could not be held. Thus, alternative educational techniques and approaches were required to deliver the curriculum remotely. Three workshops were developed to thoughtfully deliver content during the COVID-19 pandemic that aligned with the university’s social distancing parameters. These workshops delivered different content to medical students at various stages of medical school. Therefore, we set out to deliver and evaluate three different approaches for delivering POCUS educational content to medical students as social distancing precautions evolved throughout the COVID-19 pandemic. The purpose of this paper is to describe each of these three unique approaches to teaching POCUS, students’ perception of each approach, and their effectiveness in accomplishing educational objectives.

## Materials and methods

This was a prospective study assessing three different methods of delivering POCUS education throughout a pandemic to 660 medical students at various stages of their medical and POCUS training. It was conducted at a large medical school with approximately 360 students per year. Workshops occurred from April 2020 to August 2020 and data was collected during that time. This study was deemed exempt by the Institutional Review Board with a waiver of informed consent.

Methods of delivering content included a remote video conferencing webinar, remote POCUS simulation, and a “progressive-dinner” style hands-on session, intentionally formatted in this manner to prevent the spread of COVID-19. All sessions, including hands-on sessions, were taught by ultrasound fellowship-trained emergency medicine faculty. In-person hands-on sessions were also taught with diagnostic radiology faculty and sonography students.

Workshop one

**Figure 1:**
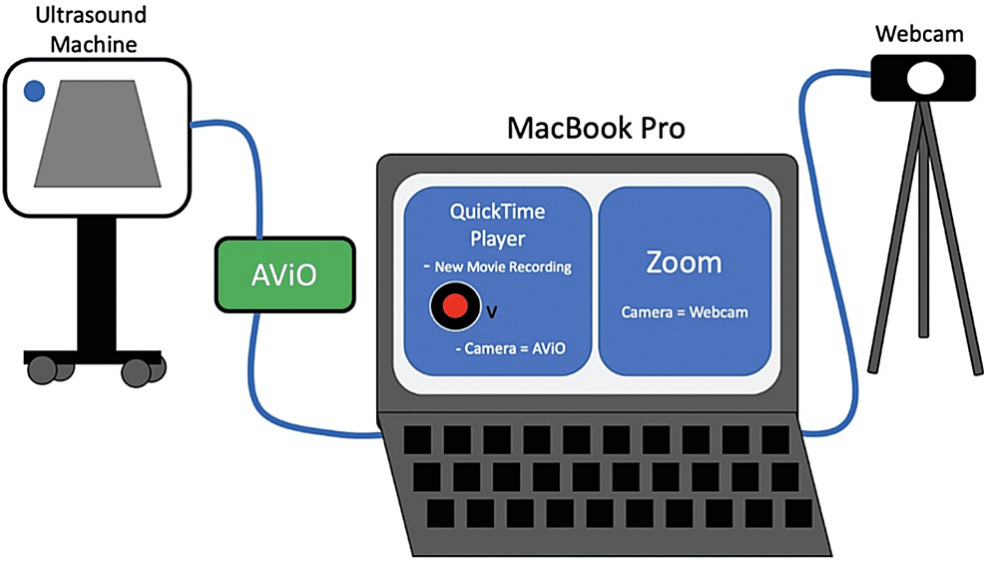
Ultrasound machine and webcam set-up The combination of webcam, PowerPoint presentation, and live-streamed images from the ultrasound involved were used for this webinar. The ultrasound machine was connected to a laptop computer via an HDMI cord to an AViO video capture device that was further connected to the laptop computer with a USB cord. The real-time ultrasound feed was delivered using Quick Time Player on the laptop computer. The computer was also connected to the webcam and this live feed was delivered utilizing Zoom video-conferencing technology. The case-based PowerPoint presentation was intermittently displayed throughout the presentation via Zoom video-conferencing technology.

During the first workshop, fourth-year medical students watched an online case-based webinar using videoconferencing technology. This session lasted 90 minutes. The objectives of this session were to understand probe placement, image acquisition, and interpretation for a focused cardiac, focused assessment with sonography in trauma (FAST), lung, and abdominal aorta POCUS exam. Cases were introduced via a PowerPoint presentation. After the case introduction, a webcam was used to demonstrate probe placement and movement while being performed on a standardized patient. The indicated views for the cases were demonstrated and image acquisition and troubleshooting techniques were discussed. While demonstrating probe placement using the webcam feed, the ultrasound images were simultaneously streamed in real-time. Learners were able to visualize how to probe manipulation changed the ultrasound images obtained concurrently via a picture in picture format (Figure 1). Prior to the webinar, participants were sent instructions and encouraged to make “homemade probes” to simulate probe placement and movement themselves while viewing the webinar at home. POCUS image interpretation and clinical integration were then discussed. There was a total of eight cases. They covered the following pathologic conditions: pneumonia, cardiac tamponade, pneumothorax, abdominal aortic aneurysm, pulmonary embolism, ectopic pregnancy, acute heart failure, and sepsis. At the conclusion of the webinar, students were asked to respond to a post-survey that consisted of a Likert scale and open-ended questions to evaluate attitudes and feedback regarding the session (Table 1).

**Table 1:** Qualitative evaluation of sessions Q&A - Question and Answer, PPT - PowerPoint

	Totals Coded Responses	% of Responses
Session 1		
Add opportunities for interaction via polls/chat/Q&A	2	1%
Add Sonogames	1	1%
Adjust time: too long to focus	3	2%
Audio issues	2	1%
Beneficial case-based session	8	6%
Beneficial practice	1	1%
Better video quality	3	2%
Difficult to ascertain exact placement of probe in video	2	1%
Difficult to learn virtually	1	1%
Effective comprehensive review	20	15%
Images referencing exact probe placement/labels	2	1%
Need for expansion of scope and references	1	1%
Needs in-person hands-on practice	22	16%
No change	8	6%
Repetition was very helpful in retaining information	2	1%
Successful virtual presentation	51	38%
The quiz did not show up with any questions	1	1%
Upload resources for future reference	2	1%
Would be beneficial to add basic ultrasound as prerequisite	2	1%
Session 2		
Great Class	49	27%
Innovative with simulation	1	0.5%
Well done: Case based format was great	4	2%
Well done: real time demonstration on patient was great	1	0.5%
Well done: Ultrasound demonstration was very helpful	3	2%
Well organized	6	3%
Well organized: Good visuals	2	1%
Well organized: informative and engaging	26	14%
Well organized: Interactive and engaging	68	37%
Well organized: no suggestions for improvement	22	12%
Constructive/Improvement Response Themes		
Confused about whether the images shown were part of the cases	1	7%
Facilitators need to be more kind/patient with Zoom regarding getting high response rate on polls	1	7%
More information about types of shock	1	7%
More ultrasound views	1	7%
Slow play ultrasound images	1	7%
Speaker could orient us to the position of the ultrasound each time	1	7%
Technology issues: internet	1	7%
Technology: difficult on laptop	1	7%
Technology: poor video/Image quality through Zoom	4	27%
Technology issues: Set up of canvas was confusing	1	7%
Ultrasound material did not translate well to online. Keep in person	1	7%
Keep ultrasound image active while answering assessment questions	1	7%
Session 3		
Great session	33	75%
Additional time for hands-on session	8	18%
Too many videos	1	2%
Additional time for equipment handling	1	2%

Workshop two

The second session utilized the same video-conferencing technology and web-cam set-up as previously described and was taught a few months later to a new class of fourth-year medical students. This session integrated several simulated patient scenarios, where instructors role-played the patient and physician encounter to better demonstrate how POCUS is used clinically within an acute care setting. As was demonstrated in the first session, a side-by-side split screen of a live ultrasound image and probe movements was broadcast to learners. This session lasted 90 minutes. Learning objectives included understanding probe placement, image interpretation and clinical utility of a Rapid Ultrasound in Shock and Hypotension (RUSH) exam.

The session included three simulated cases, each of a different patient presenting acutely for undifferentiated hypotension. The approach to a patient with undifferentiated hypotension, including the use of POCUS to aid in diagnosis was discussed. Cases for this session included hemorrhagic shock from a splenic laceration, ruptured abdominal aortic aneurysm, and sepsis secondary to an infected, obstructed ureteral stone. At the conclusion of each case clinical management was discussed, as was POCUS image interpretation, diagnosis, and treatment.

Polling questions were developed to encourage learner participation and were asked during each patient scenario. Students were asked a total of 12 multiple-choice or true/false questions. These questions were designed to assess students’ prior experience with POCUS, knowledge of ultrasound image acquisition, ultrasound interpretation, and clinical integration (Table 2). Student responses to the polling questions were recorded. A post-session survey assessed students’ attitudes and feedback regarding the workshop (Table 1). Responses from in-session polling questions to post-session responses were subsequently compared (Table 3).

**Table 2:** Simulated case (session 2) knowledge questions and responses RUSH - Rapid Ultrasound in Shock and Hypotension, LV - Left Ventricular, E-FAST - Extended-Focused Assessment with Sonography in Trauma, IVC - Inferior Vena Cava

	Correct	Responses	Total Respondents
Acquisition			
What views are included in the RUSH exam?	177 (96%)	184 (74%)	247
Where and in what direction would you place the probe and indicator to obtain a Morison’s pouch view?	72 (35%)	207 (63%)	326
Where and in what direction would you place the probe and indicator to obtain a long axis view of the right kidney?	165 (83%)	200 (61.3%)	326
Interpretation			
Identifying normal LV ejection fraction	39 (25%)	153 (49%)	313
Recognize free fluid in the left upper quadrant on E-FAST	80 (39%)	207 (63%)	326
Identify which part of the renal anatomy to evaluate for hydronephrosis	179 (81%)	221 (66%)	334
Recognize hydronephrosis	141 (67%)	210 (63%)	334
Recognize a normal abdominal aorta	88 (44%)	200 (60%)	334
Identify the size cut-off for a normal caliber aorta	130 (60%)	217 (63%)	334
Clinical Integration			
Recognize a flat IVC, intravascular volume depletion and need for volume resuscitation	187 (95%)	197 (62%)	320
Recognize the need for operative intervention in a hypotensive patient with a positive E-FAST	166 (75%)	220 (67%)	326
Recognize the need for operative intervention in a patient with an infected ureteral stone (hydronephrosis and infected urine)	34 (18%)	210 (63%)	334

**Table 3:** Simulated case (session 2) comparing in-session responses to post-session responses E-FAST - Extended-Focused Assessment with Sonography in Trauma, AAA - Abdominal Aortic Aneurysm, IVC - Inferior Vena Cava

	Pre	Post	p-value
Acquisition			
Where and in what direction would you place the probe and indicator to obtain a long axis view of the right kidney?	165/200 (83%)	337/340 (99%)	<0.001
Interpretation			
Recognize free fluid on E-FAST	80/207 (39%)	335/340 (99%)	<0.001
Identify which part of the renal anatomy to evaluate for hydronephrosis	179/221 (81%)	338/340 (99%)	<0.001
Recognize the presence or absence of hydronephrosis	141/210 (67%)	271/340 (80%)	<0.001
Recognize a normal abdominal aorta versus AAA	88/200 (44%)	337/340 (99%)	<0.001
Identify the size cut-off for a normal caliber aorta	130/217 (60%)	334/340 (98%)	<0.001
Clinical Integration			
Recognize a flat IVC, intravascular volume depletion and need for volume resuscitation	187/197 (95%)	336/340 (99%)	0.014
Recognize the need for operative intervention in a patient with an infected ureteral stone (hydronephrosis and infected urine)	34/210 (18%)	302/340 (89%)	<0.001

Workshop three

As the pandemic progressed and social distancing precautions lessened, the third session reverted to a combination of online didactics and in-person hands-on scanning. To comply with precautions, a “progressive-dinner” format was used to provide hands-on instruction to second year medical students in association with their physical exam course. Before participating in the workshop students completed a pre-survey and knowledge assessment and were expected to watch three short POCUS videos that covered the cardiac, FAST, abdominal aorta and biliary exams. Each video covered indications for the exam, briefly discussed how to perform the exam and reviewed normal and common pathologic findings.

For the workshop, students were scheduled to arrive in 10-minute time increments. After checking in for the session, students watched an educational video that demonstrated how to operate the handheld POCUS machine and how to obtain all images of the POCUS exam they were about to perform. A QR code was posted outside the designated scanning room where students were expected to watch the video on their own smart device. The first video reviewed ultrasound probe orientation, the FAST, renal and gallbladder exams (Video 1). After watching this short video, students then entered the scanning room and performed a FAST, renal and gallbladder exam previously described in the educational video on a standardized patient while an instructor stood by and provided direct one-on-one instruction and feedback. After completing the exam, learners would then progress to the next learning station and repeat the process for a different exam type. The second video and hands-on sessions covered the cardiac and abdominal aorta exams (Video 2). In total, each hands-on session and each video was timed to be 10-minutes in length. For this workshop, there were two videos and two hands-on sessions. The learning objectives for this session included review of ultrasound orientation, understanding probe placement and image acquisition for performing a gallbladder, FAST, renal, cardiac, and abdominal aorta POCUS exam. At the conclusion of the workshop students completed a post-survey and knowledge assessment.

**Video 1:** Probe orientation, FAST, renal, and gallbladder exams FAST - Focused Assessment With Sonography in Trauma

**Video 2:** Cardiac and abdominal ultrasound

## Results

Data analysis

Quantitative and qualitative results were analyzed for the three different workshops. Differences between pre- and post-knowledge responses were analyzed using Chi-Square. A 5% significance level was used for all tests. Quantitative analyses were performed using Microsoft Excel (Microsoft, Redmond, WA) and VassarStats (http://vassarstats.net, Poughkeepsie, NY). Qualitative responses were analyzed using the structural coding approach, and a descriptive method to identify emerging themes within the responses [7].

Session 1: webinar results

One hundred and eighty fourth year medical students participated in session 1; 178 (98.9%) responded to a post-survey. Of 178 students, 177 (99%) respondents found the session well prepared, organized, intellectually challenging, and stimulating. All students indicated that they learned something in this session and that it was relevant and applicable to clinical practice. Ninety-nine percent (177) of students indicated that the instructors facilitated an engaging and positive learning environment.

Ninety (50%) students provided qualitative feedback on the webinar session; 34 (38%) respondents stated it was a successful virtual presentation, 14 (15%) found the content to be an effective comprehensive review, five (6%) stated the case-based session was beneficial, and another five (6%) suggested no change to the session. Fourteen (16%) respondents emphasized the need for hands-on practice and stated that this was an essential element that was missing from the virtual format (Table 1).

Session 2: simulated case session results

Three hundred and forty-four fourth year medical students participated in session two: 42% of students self-reported little to no prior experience with ultrasound, while most students had some prior POCUS experience.

Three-hundred and forty (99%) students provided feedback on session 2, and 99% indicated that they learned something during this session that was applicable to clinical practice. Ninety-nine percent of students found the session well prepared, organized, intellectually challenging and stimulating, and similarly 99% indicated that the instructors facilitated an engaging and positive learning experience.

In-case questions were aimed at image acquisition (3), image interpretation (6) and clinical integration (3), see Table 2. For acquisition, 96% (177/184) of students knew the views of the RUSH examination. Only 35% (72/207) of students were able to correctly identify where to position the ultrasound probe and direction of the indicator when performing a Morison’s pouch view of the E-FAST exam to identify free intraperitoneal fluid, while 83% (165/200) of students were able to correctly identify where to position the ultrasound probe and direction of the indicator when performing a right kidney long axis view to assess for hydronephrosis.

For image interpretation, students were asked to interpret a normal parasternal long axis cardiac view and 25% (39/153) of students recognized this video as normal. Other students misclassified cardiac images as having evidence of pericardial effusion, tamponade or left ventricular systolic dysfunction. Only 39% (80/207) of students recognized free intraperitoneal fluid on the left upper quadrant view of on an E-FAST exam. Most students, 81% (179/221) were able to identify which part of the kidney to evaluate for hydronephrosis and 67% (141/210) recognized moderate hydronephrosis on a video. Forty-four percent (88/200) and 60% (130/217) of students were able to recognize a normal caliber abdominal aorta and identify the correct size cut-off, respectively.

For clinical integration, 95% (187/197) of students were able to recognize a flat inferior vena cava on POCUS in a hypotensive patient concerning for depleted intravascular volume and the need for volume resuscitation with IV fluids. Most students, 75% (166/220) were able to recognize a positive E-FAST with intraperitoneal blood in a hypotensive patient who was not responding to volume resuscitation with blood and needed emergent operative intervention. Only 18% (34/210) of students correctly identified that a patient needed to go to the operating room for an infected ureteral stone as evident by moderate hydronephrosis and a urinalysis positive for infection, instead of being discharged or admitted to the floor. A post-session survey was completed by 340 students and found significant improvement in knowledge regarding image acquisition, interpretation, and clinical integration, see Table 3.

One hundred and ninety-one (55%) students provided qualitative feedback on the simulated case session; 95% (182/191) had a positive response about the session; 3% (6/191) of respondents provided suggestions for further improvements and 5% (9/191) provided constructive comments, which were technology related (Table 1).

Session 3: progressive dinner results

One hundred and thirty-two second year medical students participated in session 3. Prior to the session 126 (95%) students had no or only limited hands-on ultrasound experience. One hundred and eight students (86%) responded to a post-session survey; 98% (106/108) felt their comfort with performing a FAST ultrasound significantly improved, two (2%) had no change and no student felt their comfort worsened. One hundred and five students (97%) felt their comfort with performing a four-view cardiac ultrasound significantly improved, three (3%) had no change and no student felt their comfort worsened. When evaluating students comfort level participating in a hands-on session despite an ongoing pandemic, 92 (85%) felt comfortable, 14 (13%) neutral and two (2%) felt uncomfortable. One hundred and seven (99%) students found this to be a quality and informative session, while one (1%) was neutral. Comparing pre- to post-session knowledge (test) responses, we found significant improvement in students’ ability to identify a pericardial effusion 46% to 69% (p<0.001) on a parasternal long axis cardiac image, and significant improvement with identifying the proper probe location and indicator direction for obtaining a subxiphoid view 54% to 85% (p<0.001).

Qualitative analysis of student feedback found 44 of 108 participants provided comments regarding the session and barriers to learning POCUS. Seventy-five percent (33/44) of students identified no barriers. Themes that emerged centered around time, equipment handling, and video structure. Eighteen percent of students indicated that they wished they had more time practicing hands-on POCUS use, or additional time to review POCUS videos. Two percent felt that there were too many videos. An additional 2% felt they would have benefited from additional time for equipment orientation.

## Discussion

POCUS education requires both knowledge-based and procedural skill-based instruction, which are often combined in the same educational experience. Conditions resulting from the COVID-19 pandemic created a challenge for providing the procedural skill-based instruction that is conventionally performed in a small, hands-on group setting. Due to social distancing guidelines surrounding the COVID-19 pandemic, this hands-on instruction was at risk of being completely lost in the undergraduate medical curriculum. In response, we created a series of educational events using a variety of readily available and affordable technologies to provide instruction on the procedural elements of POCUS examinations. In each case, student responses to the instruction and methodologies were overwhelmingly positive.

While the training was carried out due to social distancing requirements of the COVID-19 pandemic, we believe the lessons learned during these sessions provide value for post-pandemic POCUS instruction, especially where commonly cited barriers of time, distance and local expertise exist [2]. Even at institutions where barriers are less prevalent, there may be times within the curriculum when in-person instruction may be challenging due to physical distance, economic constraints, or availability of faculty where these instructional methodologies may be appropriately substituted for more traditional, resource intense, small group, hands-on instruction. Based on our experience, we believe these remote learning sessions could be used and/or adapted for a wide range of institutions and scenarios.

Our results show that learners clearly see value in instructional methodologies where observation of technical skills is demonstrated in a live videoconference format that is separated from an expectation of practice of those skills during the same session. Such observational learning has been shown to be beneficial in learning both simple and complex motor skills [8,9] In the first two sessions, fourth-year students had previously been exposed to POCUS and had received hands-on POCUS instruction in at least three of their clinical clerkships. While both webinar sessions presented a new exam, the RUSH exam [10] is composed of elements of different exams they had previously learned. While the technique to acquire images was not entirely novel to the students, the process of image acquisition, as well as the clinical scenarios, were unique to their previous POCUS use and exposure. Therefore, we suspect student perceived value, is at least partially, derived from the other components of POCUS learning [11], namely understanding the indications for using POCUS within clinical care, pathologic image recognition, and integration of POCUS exam findings into the clinical case presented.

To improve upon the delivery of the material by increasing interaction and to assess students’ baseline knowledge of specific POCUS exams, we created a series of questions that we asked before each case during the second online seminar. In doing so, we learned that although students had prior exposure to the FAST, renal, aorta, and cardiac exams, there were still large knowledge gaps in both knowing where to place the probe and distinguishing normal versus basic pathologies. This was particularly true for the cardiac exam and least likely for the renal exam. We suspect that this may be a result of the complexity of these exams, where the renal exam is about identifying a static structure as opposed to the heart which is a moving and dynamic organ that requires more time and practice to learn and understand [12].

While the third session represented a different cohort of students, at an entirely different point in their medical and POCUS education, their response to a unique approach to the delivery of POCUS instruction was equally positive, with 99% of students indicating that they found the session to be of high quality and informative. As with the webinar sessions, a unique observational learning approach was used by having students watch a demonstration of the exam on their personal smartphone before immediately going into a session to practice those skills. Based on our assessment, this combined video and unique hands-on instructional approach, was effective in meeting coarse objectives, namely, to identify proper probe placement for a subxiphoid view of the heart (54% to 85%) and to then identify a pericardial effusion (46% to 69%).

We believe these different educational strategies provide strategies to overcoming traditionally cited barriers to POCUS instruction even outside of the current COVID-19 pandemic. Access to machines and patients to scan, along with qualified instructors in the medical education landscape can be difficult given multiple barriers. While machine cost continues to decrease, resulting in more accessibility; the economics of machine acquisition can still be prohibitive. Furthermore, traditional hands-on instruction occurs in small groups with 4-5 students assigned to a single instructor. Utilizing an intentional strategy of observational learning, while not a replacement for hands-on instruction, seems to provide significant benefit and may improve efficiency in meeting the end goal of teaching students to competently perform specific POCUS exams. Maximizing the learning efficacy of a student’s hands-on probe time should be a priority within the educational experience and may help allay two commonly cited concerns of lack of faculty expertise and time within the curriculum. Learning the concepts of POCUS while providing a preliminary foundation, including hands-on instruction, before the actual hands-on lab, will likely serve to enhance the hands-on experience for both learner and instructor. More research will be needed to find the ideal combination and approach.

The COVID-19 pandemic forced medical educators to be creative and adapt to seemingly ever-changing safety parameters. While this adaptation and the educational processes developed to work during pandemic instruction are novel, the lessons learned will continue to shape the way educators provide POCUS instruction. While more study is needed given our utilization of two separate student cohorts, our results are encouraging. Overwhelmingly positive student feedback provides support for these two types of methodologic approaches to POCUS education and may inform the future instructional design to POCUS education, especially where barriers of time, distance, and expertise provide challenges to providing quality POCUS education.

Limitations

While the initial two workshops were able to demonstrate how a POCUS exam was performed, showing precise locations of probe placement and fine movements of the probe to achieve the desired images, it still did not provide a means for learners to perform the exam under direct observation by an instructor. This is an essential component of POCUS training, and this was a dominant theme in our thematic analysis. While handheld ultrasound device prices are more affordable than they were previously, they are still not so ubiquitous or affordable that students are likely to have their own device. However, it is possible that a shared pool of devices that are available to be borrowed for a limited time. Handheld devices could be used with the training, either live or recorded, to allow a learner to remotely practice scanning during the session. While we did have learning objectives for the initial session, due to time constraints and the remote learning nature of the session, we were not able to directly evaluate whether these objectives were achieved.

## Conclusions

POCUS education includes both knowledge and skill-based learning. While the ideal educational format is to combine these two facets into one shared in-person session, the above paper demonstrates that alternative educational approaches are well-accepted replacements or adjuncts to traditional learning methods. These teaching methods address limitations in hands-on learning caused by social distancing precautions during the COVID-19 pandemic and those that may occur in non-pandemic times.
